# Aerobic fitness, phase angle, and bioelectrical impedance vector analysis in adolescents living with HIV: a cross-sectional study

**DOI:** 10.1590/1984-0462/2025/43/2024277

**Published:** 2025-07-28

**Authors:** Marcos Cezar Pitombo da Silva, Diego Augusto Santos Silva, Priscila Custódio Martins, Nassib Bezerra Bueno, Fabiana Andréa Moura, Luiz Rodrigo Augustemak de Lima

**Affiliations:** a Universidade Federal de Alagoas, Maceió, AL, Brazil.; b Universidade Federal de Santa Catarina, Florianópolis, SC, Brazil.; c Universidade do Extremo Sul Catarinense, Criciúma, SC, Brazil.

**Keywords:** Acquired immunodeficiency syndrome, Adolescent health, Bioelectrical impedance, Body composition, Cardiorespiratory fitness, Síndrome da imunodeficiência adquirida, Saúde do adolescente, Impedância elétrica, Composição corporal, Aptidão cardiorrespiratória

## Abstract

**Objective:**

To analyze the association between phase angle (PhA) and bioelectrical impedance vector analysis (BIVA) with aerobic fitness in HIV+ adolescents.

**Methods:**

Aerobic fitness was assessed using the modified Canadian Aerobic Fitness Test, PhA, and BIVA by bioelectrical impedance analysis. Multivariable linear regression was performed to test the association between peak oxygen consumption (peak VO_2_) and PhA. BIVA ellipses were evaluated considering a reference population of Spanish adolescents.

**Results:**

Forty-seven HIV-infected adolescents (10–18 years old) were included; 25 (53%) were females. Mean peak VO_2_ and PhA were 48.9 standard deviation (±) 7.0 ml.kg^-1^.min^-1^ and 4.6±1.4 degrees, respectively. Multivariable analysis revealed a significant positive association between peak VO_2_ and PhA (β_std_=0.349; p=0.025). Peak VO_2_ adjusted for covariates explained 49% of PhA variation. BIVA ellipses indicated lower classifications for body cell mass (BCM) and fatfree mass (FFM) in HIV+ adolescents (*vs* reference population) (p<0.001). HIV+ females (*vs* HIV+ males) showed a higher classification for dehydration and a lower classification for BCM (p=0.007). No relevant differences were found between the subgroups based on aerobic fitness (p=0.735).

**Conclusions:**

A significant association exists between peak VO_2_ and PhA in HIV+ adolescents. Alterations in hydration and lower classifications for BCM and FFM were observed in this population.

## RESUMO

**Objetivo:**

Analisar a associação do ângulo de fase (AnF) e da análise vetorial de impedância bioelétrica (BIVA) com a aptidão aeróbica em adolescentes que vivem com HIV.

**Métodos:**

A aptidão aeróbica foi avaliada pelo Teste Canadense Modificado de Aptidão Aeróbica, AnF e BIVA por meio da análise de impedância bioelétrica. Foi realizada regressão linear multivariada para testar a associação entre o consumo de oxigênio de pico (VO_2_ pico) e o AnF. As elipses da BIVA foram avaliadas considerando uma população de referência de adolescentes espanhóis.

**Resultados:**

Quarenta e sete adolescentes infectados pelo HIV (10–18 anos) foram incluídos; 25 (53%) eram do sexo feminino. A média do VO_2_ pico e do AnF foram 48,9 desvio padrão (±) 7,0 ml.kg^-1^ .min^-1^ e 4,6±1,4 graus, respectivamente. A análise multivariada revelou uma associação positiva significativa entre VO_2_ pico e AnF (β_std_ =0,349; p=0,025). O VO_2_ pico ajustado para covariáveis explicou 49% da variação do AnF. Elipses de BIVA indicaram uma classificação mais baixa para massa celular corporal (MCC) e massa livre de gordura (MLG) em adolescentes com HIV (*vs* população de referência) (p<0,001). Adolescentes com HIV do sexo feminino (*vs* masculino) apresentaram classificação mais alta para desidratação e mais baixa para MCC (p=0,007). Não foram encontradas diferenças relevantes entre os subgrupos com base na aptidão aeróbica (p=0,735).

**Conclusões:**

Existe uma associação significativa entre VO_2_ pico e AnF em adolescentes com HIV. Observou-se alterações na hidratação e classificação mais baixa para MCC e MLG.

## INTRODUCTION

 The advent of antiretroviral therapy (ART) has considerably increased the life expectancy and improved the quality of life of individuals living with HIV (HIV+). Long-term ART use is associated with several morphological and physiological adverse effects, including body fat (BF) redistribution, muscle mass loss, and reduced aerobic capacity.^1,2^ Additionally, HIV+ children on ART exhibit lower total body water levels (both intracellular and extracellular) compared to their HIV-negative peers.^3^ Furthermore, HIV+ individuals are at a higher risk of developing cardiovascular complications due to the exacerbation of inflammatory, metabolic, and cardiovascular markers.^4^

 Aerobic fitness is a predictor of health indicators in adolescents and is expressed as oxygen consumption (VO_2_).^5,6^ In adolescents, peak VO_2_ is widely used as a physiological marker of aerobic fitness, representing the highest VO_2_ rate achieved during exercise. This parameter is particularly suitable for adolescents due to their low motivation and difficulty sustaining maximal effort.^5^ Various protocols are available to assess aerobic fitness; however, submaximal protocols are a practical alternative for HIV+ adolescents in outpatient settings, as they are cost-effective, easy to administer, and safe.^6^

 Given the deleterious effects of HIV and long-term ART, the assessment and monitoring of body composition (BC) parameters are essential.^1^ Bioelectrical impedance analysis (BIA) is a cost-effective option for outpatient settings and offers a validated, non-invasive, easy-to-apply, and reproducible method for evaluating BC in HIV+ individuals.^7,8^ Several approaches exist for assessing BC using BIA, with single-frequency tetrapolar bioelectrical impedance analysis (SF-BIA) at 50 kHz being the most widely used. This method relies on standardized electrode placement on the wrist and ankle.^9^ However, BC estimation using BIA depends on predictive equations, which may limit its applicability.^10^ To address these limitations, alternative parameters such as phase angle (PhA) and bioelectrical impedance vector analysis (BIVA) can be utilized.^10^

 PhA is recognized as an indicator of cellular health and has been applied across various clinical conditions.^9-11^ BIVA enables the identification and classification of nutritional status and the monitoring of hydration levels through the analysis of ellipse axes.^12^ Both PhA and BIVA serve as valuable markers of critical health status.^10,11,13^ Previous studies have examined the relationship between PhA and physical fitness in HIV+ adolescents,^11^ as well as PhA and BIVA in healthy adolescents.^12,14^ These studies demonstrate associations between PhA, BIVA, and key health indicators, such as aerobic fitness and BC.^12,14^ Incorporating PhA and BIVA into the continuous assessment of nutritional status in HIV+ adolescents may enhance diagnostic accuracy and inform therapeutic decision-making. 

 The present study aimed to test the association between aerobic fitness, PhA, and BIVA in HIV+ adolescents. We hypothesized that HIV+ adolescents would exhibit low levels of aerobic fitness, PhA, hydration, and body cell mass (BCM). Additionally, we expected PhA and BIVA to be directly associated with aerobic fitness. 

## METHOD

 This observational cross-sectional study is part of the "PositHIVa health of adolescents from Alagoas: monitoring of lifestyle, physical fitness, cognition and cardiometabolic risk" project. 

 The study was conducted between March 2022 and December 2023 at the Specialized Care Service (SAE) in HIV/AIDS of the Dr. Helvio José de Farias Auto Teaching Hospital (HEHA), city of Maceió, state of Alagoas, Brazil. 

 Participants were eligible for inclusion if they met the following criteria:

1. Aged 10–18 years;
2. Received care at SAE-HEHA;
3. Had a confirmed HIV diagnosis recorded in their medical records; and
4. Had available clinical and outpatient data.

The exclusion criterion was the inability to stand or move independently. 

 A Tetrapolar Sanny® SF-BIA device (model BIA1010) was used for BIA analysis. The device was calibrated by the manufacturer prior to the study and by the evaluator before each assessment. A disposable electrode (ECG), MSGST-06 model for adults/infants, was utilized. A test-retest comparison with Sanny’s proprietary electrode showed no significant differences between electrode models. Before undergoing SF-BIA, participants were screened to ensure compliance with pre-test guidelines.^15^ For standardization of the SF-BIA technique, sensory electrodes (proximal and distal) were positioned on the metacarpophalangeal and metatarsophalangeal joints, respectively. Measurements were taken on the right side of the body while participants lay in the supine position on a non-electrically conductive surface. The skin at the electrode placement sites was cleaned with alcohol, and the arms and legs were positioned at an angle of approximately 30 to 45° from the trunk, following established protocols.^15^ The procedure involved three automatic measurements, with the average value used for analysis. 

**Figure 1:**
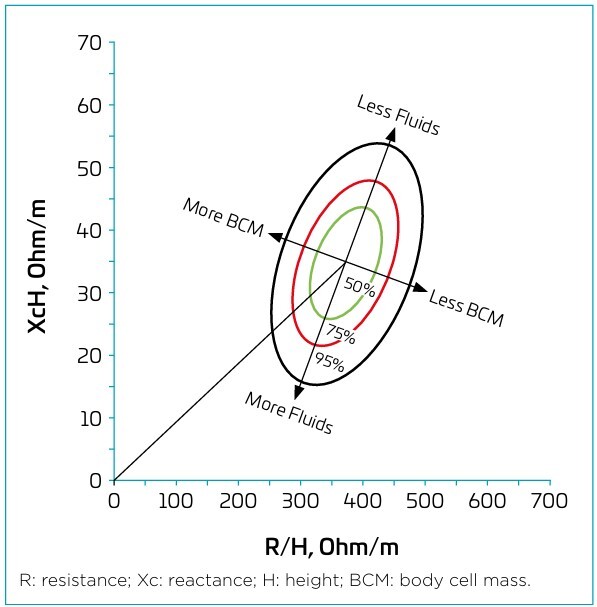
Graphical representation and interpretation of bioelectrical impedance vector analysis.^16^

 Resistance (R) and reactance (Xc) were obtained using SF-BIA of 50 kHz^10^ to calculate the PhA, using the formula: $\frac{Xc}{R}x\frac{180{}^{\circ}}{\pi }$ . A PhA value below 5.0 degrees is indicative of inflammation, cellular dysfunction, and damage, whereas value above 5.0 degrees is associated with better health status and a reduced risk of mortality.^10,11^ Impedance vectors were calculated and analyzed using the BIVA Software 2002®.^16^ R and Xc values were standardized by participants’ height and incorporated into an elliptical analysis model, expressed in ohm/meter (Ω/m) (Figure 1).^17^ The resulting vector was compared against tolerance ellipses, representing the 50% (median), 75% (third quartile), and 95% (95th percentile) percentiles of a reference population. Additionally, confidence ellipses were used to indicate the 95% probability of locating the true mean.^10,16,17^

 The vectors, represented on the RXc graph, enable the analysis of soft tissues based on their electrical properties, without requiring prior knowledge of body weight.^16,17^ Vectors outside the 75% tolerance ellipse indicate altered tissue impedance, with long vectors outside the upper pole suggesting dehydration, and short vectors outside the lower pole indicating hyperhydration. Displacement of the vector along the minor axis to the left, or along the major axis to the right, reflects variations in BCM and fat-free mass (FFM) (Figure 1).^16^ Healthy Spanish adolescents aged 14–19 years were selected as the reference population^18^ since they fall within the age range of the participants in the current study, and due to the lack of reference values specific to HIV+ adolescents. 

 The modified Canadian Aerobic Fitness Test protocol involved a step-up and step-down exercise, initially using two 20.3 cm steps and later progressing to a single 40.6 cm step. The cadence was set according to the Canadian Society for Exercise Physiology’s guidelines, with the test conducted in progressive stages of three minutes each. Adolescents began the test at the stage designated by their sex and age, with males starting at stage 4 and females at stage 3.^19^ Upon reaching more advanced stages (7 and 8 for males, stage 8 for females), only one 40.6 cm step was used (positioned either at the back or side of the bench).^19^ The researcher demonstrated the test procedure and allowed a familiarization period before starting. The test was terminated when the adolescents reached 85% of their predicted maximum heart rate, calculated using the formula 220 – age, or in cases of voluntary withdrawal.^19^ Heart rate was monitored using a Polar® S601i heart rate monitor (Polar, Finland), with a strap worn around the participants’ chest. The final stage reached by each subject was recorded. Peak VO_2_ (ml.kg^-1^.min^-1^) was estimated using the following equation: 17.2+(1.29×O_2_ consumption stage) – (0.09 × weight in kg) – (0.18 × age in years).^19^ Aerobic fitness was classified as either inadequate or adequate based on age- and sex-specific cut-points, with the Z-score serving as an indicator of cardiometabolic risk in adolescents.^20^

 Body mass (kg), height (cm), and skinfolds thicknesses (subscapular, abdominal, triceps, and calf) were measured following the International Society for the Advancement of Kinanthropometry protocol.^21^ The following devices were used for the assessments: a) Cescorf® pocket stadiometer (Cescorf Ltda., Porto Alegre, Brazil); b) Tanita® electronic scale (BF683W, Arlington Heights, IL, USA); c) Cescorf® inelastic anthropometric tape (Cescorf Ltda., Porto Alegre, Rio Grande do Sul, Brazil), and d) Lange® skinfold caliper (Beta Technology, Santa Cruz, CA, USA). FFM was estimated applying an equation validated for healthy children and HIV+ adolescents: (3.74–0+0.459*height^2/R+0.064*body mass)/(0.769–0.009*age–0.016*sex).^7^ BF percentage was calculated as follows: %BF=-10.35622 + (subscapular skinfold*0.6324226)+(∑4 skinfolds*0.2356916) + (abdominal skinfold*–0.2812848) + (sex*–1.538853) + (stature*0.0664786).^22^ The decision to calculate BF by anthropometric methods was made to avoid using the same raw SF-BIA data (R and Xc, and consequently PhA). Using the same raw data would introduce an artificial correlation into the regression analysis, as BF was used as a covariate. However, it is important to note that anthropometry and bioimpedance are not completely independent methods, as both rely on the assumption of constant hydration in lean body mass. 

 A semi-structured questionnaire was used to collect sociodemographic data (age, sex, self-declared skin color, and guardians’ income), as well as information on physical activity and screen time behaviors. The age range of 10–18 years was selected to encompass the largest group of adolescents treated at the reference hospital. The physical activity questionnaire for older children (PAQ-C), which is validated for HIV+ adolescents, was used to assess physical activity.^23^ It consists of nine questions, with the final score considered in the analysis. Screen time was assessed using questions from the PeNSE Study, focusing on the time spent watching television and using mobile phones, tablets, and laptops.^24^

 Sexual maturity was assessed through self-evaluation using images corresponding to the Tanner pubertal stages of genital and breast development.^25^ Participants were guided by researchers of the same sex to minimize any discomfort. This self-assessment scale has demonstrated strong validity in the Brazilian population.^26^

 The duration of ART in years, CD4+ T lymphocyte count (cells per mm^3^), and HIV RNA viral load (copies/ml) were retrieved from the medical records. 

 The study was approved by the Ethics Committees for Research Involving Humans of the State University of Health Sciences of Alagoas (no. 4.564.290) and of the Federal University of Alagoas (no. 4.506.466). Guardians and participants provided informed consent by reading and signing the form, agreeing to participate in the study. 

### Statistical analysis

 Descriptive statistics were calculated, including mean, standard deviation, and both relative and absolute frequencies. Kurtosis and skewness were examined to assess data normality, supplemented by histograms and the Shapiro-Wilk test. The independent Student’s *t*-test, Mann-Whitney U test, and Pearson’s chi-square test were used to evaluate differences between sexes. 

 In BIVA, differences in confidence ellipses were tested. The R and Xc values were standardized by height (R/height and Xc/height). Hotelling’s t^2^ test was used to compare population vectors. For the tolerance ellipses, the R/height and Xc/height values were plotted individually and compared with reference values stratified by sex. Additionally, subgroup analyses were performed among HIV+ adolescents based on aerobic fitness (adequate *vs* inadequate) and sex (male *vs* female). Aerobic fitness, like BC, is a crucial health indicator, and subgroup analysis aimed to investigate whether adolescents with inadequate aerobic fitness exhibited poorer indicators than those with adequate fitness.^2^ Since BC tends to vary by sex, employing individual references was considered essential. The sex-based analysis further aimed to explore potential differences within the study population.^12^

 Pearson and Spearman correlation analyses were conducted to assess the relationships between the independent variable (peak VO_2_) and covariates (sex, age, sexual maturity, BF, physical activity, screen time, CD4+ T lymphocytes, viral load, ART regimen, and ART duration) with the dependent variable (PhA). Multivariable linear regression was employed to examine the association between PhA and peak VO_2_. Two models were adjusted for covariates based on existing literature and factors influencing PhA.^11,13,27^ Model 1 was adjusted for peak VO_2_, physical activity, CD4+ T lymphocytes, viral load, ART regimen, and sexual maturation. Model 2 was adjusted for peak VO_2_, physical activity, CD4+ T lymphocytes, viral load, ART regimen, sexual maturation, and sex. Model diagnostics were performed applying the variance inflation factor (VIF), Akaike information criterion (AIC), and Bayesian information criterion (BIC). The regression coefficients (β), 95% confidence intervals (CI), coefficient of determination (R^2^), significance of the regression model (p*), and effect size (Cohen’s f^2^) were estimated. All analyses were performed using the Stata® 13.0 statistical software, considering an alpha value of 5%. Furthermore, a post hoc statistical power analysis was conducted using G*Power 3.1.9.7, indicating a power (β) of 99% for a sample size of 47 participants, including six predictors, based on the R2 value from the multivariate regression analyses. 

## RESULTS

 The management of the SAE reported that 72 HIV+ adolescents were treated at the outpatient clinic during 2022 and 2023. Patients who could not be located (n=1), those who declined to participate in the study (n=20), and those unable to stand and/or move (n=4) were excluded. Forty-seven HIV+ adolescents were included in the present study (25 females, 53%) (Table 1). Missing data were observed for physical activity assessment, BF estimation, and SF-BIA tests (n=3); aerobic fitness (n=2); and sexual maturation stage and CD4+ T lymphocyte count (n=1). The main reasons for missing data included the timing of medical appointments, difficulty obtaining medical records, and transportation issues. Male adolescents exhibited higher FFM, higher peak VO_2_, lower sum of skinfolds, lower BF, and lower resistance than females. PhA values below 5.0 degrees were observed in 28 participants, with higher rates in females (n=17). 

**Table 1:** Clinical and behavioral characteristics of adolescents living with HIV in Maceió (AL), Brazil, stratified by sex, 2023.

Variables	Total sample (n=47)	Female (n=25)	Male (n=22)	t/U	p-value
		Mean (SD)			
Age (years)	14.36 (2.22)	14.40 (2.32)	14.31 (2.14)	0.12	0.901
Body mass (kg)	46.49 (12.39)	45.18 (11.56)	47.97 (13.39)	-0.62	0.536
Height (cm)	156.50 (10.25)	154.31 (8.90)	158.98 (11.28)	-1.58	0.121
Z-score Height/Age	-0.72 (1.15)	-0.63 (1.08)	-0.83 (1.24)	0.59	0.561
Z-score BMI/Age	-0.64 (1.40)	-0.59 (1.15)	-0.70 (1.67)	0.27	0.796
Sum of skinfolds (mm)*	52.82 (24.31)	58.70 (22.86)	46.95 (24.79)	2.23	0.026^†^
FFM (kg)^‡^	26.96 (6.11)	23.87 (3.85)	30.2 (6.43)	-4.02	<0.001^†^
BF (%)*	14.27 (7.18)	16.10 (7.13)	12.43 (6.91)	2.34	0.019^†^
Screen time (h/week)	5.57 (2.34)	5.60 (2.58)	5.54 (2.10)	0.08	0.938
Physical activity (score)*	1.80 (0.73)	1.66 (0.71)	1.94 (0.74)	-1.27	0.211
Peak VO_2_ (ml.kg^-1^.min^-1^)^§^	49.01 (7.06)	45.92 (6.13)	52.09 (6.68)	-2.98	0.005^†^
R (Ω)^‡^	679.72 (92.38)	726.84 (76.40)	630.45 (82.40)	4.07	<0.001^†^
Xc (Ω)^‡^	53.68 (15.68)	54.46 (19.60)	52.87 (10.57)	0.43	0.666
Phase angle (degrees)^‡^	4.60 (1.39)	4.35 (1.65)	4.85 (1.04)	-1.36	0.173
ART duration (years)	9.36 (3.25)	8.91 (3.49)	9.88 (2.94)	-1.37	0.169
Viral load (copies/ml)	5278.25 (20437.08)	8484.96 (27507.18)	1634.27 (4900.65)	1.35	0.176
CD4+ (cells per mm^3^)^/^	889.97 (432.97)	830.87 (383.07)	954.45 (482.33)	-0.97	0.339
		n (%)		χ2	p-value
Skin color					
White	9 (19.15)	4 (16.00)	5 (22.73)	0.34	0.559
Black, Brown, Yellow and Indigenous	38 (80.85)	21 (84.00)	17 (77.27)		
Income					
Up to two wages	36 (78.26)	21 (84.00)	16 (71.43)	1.06	0.303
More than two wages	10 (21.74)	4 (16.00)	6 (28.57)		
ART regimen					
Without PI	19 (40.43)	13 (52.00)	6 (27.27)	2.97	0.085
With PI	28 (59.57)	12 (48.00)	16 (72.73)		
Pubertal stage^//^					
I	5 (10.87)	2 (8.00)	3 (14.29)	2.35	0.672
II	8 (17.39)	6 (24.00)	2 (9.52)		
III	12 (26.09)	7 (28.00)	5 (23.81)		
IV	14 (30.43)	7 (28.00)	7 (33.33)		
V	7 (15.22)	3 (12.00)	4 (19.05)		
Peak VO_2_ (ml.kg^-1^.min^-1^)^§^					
Inadequate	8 (19.05)	3 (14.29)	5 (23.81)	0.62	0.432
Adequate	34 (80.95)	18 (85.71)	16 (76.19)		

ART: combination antiretroviral therapy; BF: body fat; BMI: body mass index; FFM: fat-free mass; kg: kilograms; n: sample; Peak VO_2_: peak oxygen consumption; PI: protease inhibitor; R: resistance; SD: standard deviation; Xc: reactance; t/U: Student *t*-test/Mann-Whitney U; χ^2^: Pearson’s chi-square test.

For the parametric variables (age, body mass, height, height/age, BMI/age, sum of skinfolds, FFM, screen time, physical activity, peak VO_2_, R, Xc, phase angle, and CD4+), the *t*-test was applied

For the non-parametric variables (BF, ART duration, and viral load), the Mann-Whitney U test was applied.

*n=44; †p<0.05; ‡n=45; §n=42; //n=46.

 Bivariate analysis revealed a positive and moderate correlation between PhA and PAQ-C. However, no significant correlations were found between PhA and peak VO_2_ (Table 2). In the multivariable analysis, peak VO_2_ was positively and significantly associated with PhA in model 1, which included peak VO_2_, physical activity, CD4+ T lymphocytes, viral load, ART regimen, and sexual maturation. However, in model 2, which includes peak VO_2_, physical activity, CD4+ T lymphocytes, viral load, ART regimen, sexual maturation, and sex, this association was no longer statistically significant. The models explained 49% and 48% of the variation in PhA, respectively. Furthermore, model quality and fit indicators (VIF=1.37; AIC=117.43; BIC=128.89) support the selection of model 1 as the best predictor of PhA (Table 3). 

**Table 2:** Correlation between phase angle, peak VO_2_, and behavioral/clinical variables of adolescents living with HIV in Maceió (AL), Brazil, 2023.

	Correlation coefficient r (p-value)		
	Phase angle		
	Total sample (n=47)	Female (n=25)	Male (n=22)
Peak VO_2_ (ml.kg^-1^.min^-1^)	0.22 (0.159)	0.16 (0.488)	0.15 (0.510)
Age (years)	-0.03 (0.819)	-0.18 (0.398)	0.26 (0.238)
Sexual maturation (stage)*	0.08 (0.607)	-0.08 (0.727)	0.26 (0.254)
Body fat (%)*	0.05 (0.732)	-0.10 (0.650)	0.29 (0.196)
Physical activity (score)	0.46 (0.002)†	0.49 (0.029)†	0.39 (0.070)
Screen time (hours on weekdays)	0.04 (0.812)	0.01 (0.966)	0.11 (0.597)
CD4+ (cells per mm^3^)	-0.14 (0.347)	-0.20 (0.370)	-0.15 (0.496)
Viral load (copies/ml)*	-0.19 (0.213)	-0.25 (0.242)	0.04 (0.857)
ART regimen (with PI; without PI)*	-0.01 (0.964)	-0.11 (0.631)	-0.06 (0.776)
ART duration (years)*	0.09 (0.543)	-0.03 (0.897)	0.13 (0.559)

*For categorical and non-parametric variables (sexual maturation, BF, viral load, ART regimen and ART duration) Spearman’s correlation was applied; †p<0.05.

ART: combination antiretroviral therapy; PI: protease inhibitor; Peak VO_2_: peak oxygen consumption;

**Table 3:** Multivariable regression analysis of variables related to phase angle, adjusted for confounding factors, in adolescents living with HIV in Maceió (AL), Brazil, 2023.

Variables	Model 1 (R^2^=0.49)			Model 2 (R^2^=0.48)		
	β (95%CI)	Std β	p-value	β (95%CI)	Std β	p-value
Peak VO_2_ (ml.kg^-1^.min^-1^)	0.071 (0.009; 0.132)	0.349	0.025*	0.0644 (-0.000; 0.129)	0.317	0.053
Physical activity (score)	1.000 (0.445; 1.556)	0.516	0.001*	0.984 (0.420; 1.547)	0.508	0.001*
CD4+ (cells per mm^3^)	-0.001 (-0.002; -0.000)	-0.382	0.005*	-0.001 (-0.002; -0.000)	-0.383	0.005*
Viral load (copies/ml)	-0.000 (-0.000; -2.960)	-0.301	0.022*	-0.000 (-0.000; -8.320)	-0.279	0.041*
ART regimen (with PI; without PI)	-0.840 (-1.651; -0.029)	-0.287	0.043*	-0.876 (-1.703; -0.049)	-0.299	0.038*
Sexual maturation (stage)	0.396 (0.035; 0.757)	0.321	0.033*	0.397 (0.032; 0.762)	0.322	0.034*
Sex (male; female)	NA	NA	NA	0.267 (-0.544; 1.078)	0.92	0.507

Model 1 (peak VO_2_, physical activity, CD4+T lymphocytes, viral load, ART regimen, and sexual maturation) p<0.001; Cohen’s f^2^=0.96; VIF=1.37; AIC=117.43; BIC=128.89.

Model 2 (peak VO_2_, physical activity, CD4+T lymphocytes, viral load, ART regimen, sexual maturation, and sex) p<0.001; Cohen’s f^2^=0.92; VIF=1.40; AIC=118.86; BIC=131.96.

R^2^: coefficient of determination; β: beta coefficient; CI: confidence interval; Std β: standardized beta; Peak VO_2_: peak oxygen consumption; ART: combination antiretroviral therapy; PI: protease inhibitors; NA: not applicable; Cohen’s f^2^: effect size; VIF: variance inflation factor; AIC: Akaike information criterion; BIC: Schwarz Bayesian criterion.

* p<0.05.

**Figure 2:**
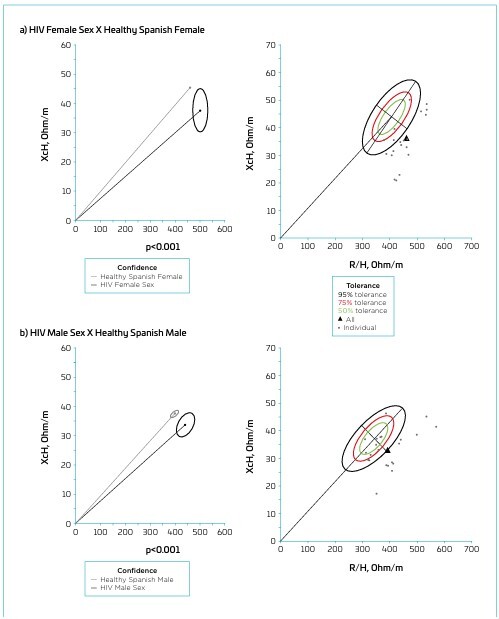
Mean impedance vectors with 95% confidence ellipses, mean impedance vectors with tolerance ellipses of 50, 75, and 95% of HIV+ adolescents in comparison to the ellipses of healthy Spanish adolescents.

**Figure 3:**
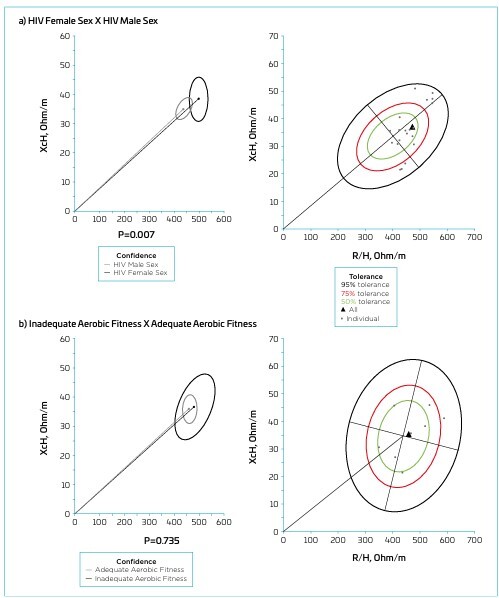
Bioelectrical impedance vector analysis — mean impedance vectors with 95% confidence ellipses and tolerance ellipses at 50, 75, and 95% of HIV+ female adolescents in comparison to the ellipses of HIV+ male adolescents, and of HIV+ adolescents with adequate aerobic fitness in comparison to the ellipses of HIV+ adolescents with inadequate aerobic fitness.

 The BIVA confidence ellipses revealed significant differences between female HIV+ adolescents and the reference population (p<0.001; Figure 2a), HIV+ adolescents and the reference population (p<0.001; Figure 2b), and between female and males HIV+ adolescents (p=0.007; Figure 3a). However, no significant differences were observed when comparing adolescents with inadequate *vs* adequate aerobic fitness (p=0.735; Figure 3b). Most HIV+ adolescents fell within the 95% tolerance ellipses compared to the reference population, indicating lower classifications for BCM and FFM. In general, females and males were in the 95% tolerance ellipses (Figures 2a and 2b), meaning both exhibited lower BCM and FFM classifications than the reference population. When comparing sexes among HIV+ individuals, most females were positioned within the 50 and 75% tolerance ellipses, suggesting similar characteristics to males, who served as the reference population. However, five HIV+ females were located within the upper 95% ellipses, indicating a higher classification of dehydration, while three were in the lower 95% ellipses, associated with reduced BCM (Figure 3c). The inadequate aerobic fitness group (*vs* adequate aerobic fitness) remained close to the center of the ellipses, indicating that the individuals are within the parameters considered normal, according to the reference population standards (Figure 3d). 

## DISCUSSION

 The main finding of this study was the direct association between peak VO_2_ and PhA in a model adjusted for physical activity, CD4+ T lymphocytes, viral load, ART regimen, and sexual maturation, explaining 49% of the variance in PhA. Additionally, BIVA analysis indicated that HIV+ adolescents exhibited lower classifications for BCM and FFM than the reference population.^18^ Further analysis within the HIV+ group revealed that females had higher classifications for dehydration and lower BCM values than males. However, no significant differences were found between HIV+ adolescents with inadequate aerobic fitness compared to those with adequate aerobic fitness. 

 HIV can reduce BCM in infected individuals, increasing their susceptibility to inflammation.^28^ A reduced BCM impairs muscle cells’ energy storage capacity, leading to decreased aerobic fitness.^11^ Consistently, our findings indicate that HIV+ adolescents exhibit lower BCM classifications. Although we initially expected inadequate peak VO_2_ values in this population, most participants demonstrated adequate levels. However, it is important to note that peak VO estimation and classification in this study were based on reference values from a population of a different nationality and without an HIV diagnosis.^20^

 Inflammation and oxidative damage can influence PhA values in individuals with hydration variations due to pathological conditions, potentially compromising the integrity and functionality of cellular membranes.^29^ Low PhA values may indicate a less favorable health prognosis, highlighting the need for targeted interventions.^9^ Studies have shown that HIV+ adults with PhA values below 5.3 and 5.6 experience lower survival rates and greater disease progression, respectively.^28,30^ Based on these thresholds, the participants in this study exhibited low PhA values. Both PhA and peak VO_2_ are critical health markers, making their clinical monitoring essential for evaluating the overall health status of HIV+ adolescents.^11,31^ Factors that influence PhA, such as sex, age, FFM, physical activity, and peak VO_2_, have been widely discussed in the literature.^11,13,27^ PhA typically increases during puberty, with a more pronounced rise in males, who experience greater gains in FFM, while females show a higher increase in BF.^27^ Physical activity improves the structure of cell membranes, leading to higher PhA values.^13^ Similar to PhA, peak VO_2_ is an important health indicator, which may explain the observed relationship between these two parameters.^11^ In this study, these factors were incorporated into two theoretical models and adjusted for confounders. Based on explanatory power, quality indicators (VIF, AIC, and BIC), and model performance, we identified model 1 (explaining approximately 49% of the variance) as the best predictor of PhA. The exclusion of sex from the final model was guided by statistical criteria. However, neither model demonstrated high explanatory power, emphasizing the value of BIVA as a complementary approach to purely statistical models in predicting PhA. 

 Accurately assessing and monitoring body integrity in pediatric patients poses significant challenges, primarily due to disease-related restrictions that hinder the application of more rigorous methods,^32^ or due to difficulties with access and acceptance by HIV+ patients. In this context, BIVA, a simple and easy-to-apply method, presents a viable option that may be better accepted by HIV+ patients. Additionally, studies have demonstrated that BIVA effectively identifies changes in nutritional status, hydration, and BCM in youth when compared to population reference levels, using tolerance ellipses.^9,12,14,32^ As such, BIVA could serve as a valuable clinical tool for monitoring BC in adolescents.^12,14^ However, there are currently no BIVA reference values specifically for Brazilian adolescents or HIV+ individuals, which limits the interpretation of data to the original reference population. Moreover, adolescents in developed countries tend to reach puberty at earlier ages compared to those in developing regions.^18^ Therefore, future studies should standardize maturation stages for BIVA analysis. Although a sample size calculation was not performed for the adolescents attending the service, the evaluated sample represented 65.3% of eligible adolescents, accounting for more than half of the potential participants. 

 Among the strengths of this study are its originality in exploring the relationship between aerobic fitness, PhA, and BIVA in HIV+ adolescents and the use of accurate and reliable instruments, particularly those that could be integrated into routine outpatient care for HIV+ patients. However, several limitations should be noted. One limitation is the heterogeneity among individuals due to differences in age, clinical conditions, and ART regimens. Another limitation is the availability of various brands and models of BIA devices, which can lead to discrepancies in measurements, potentially affecting the results.^9,33^ This underscores the need for standardization in manufacturing these devices. Moreover, the lack of validation studies for BIA in pediatric HIV+ populations, in comparison to reference techniques, presents a further challenge.^3^ Due to the absence of PhA and BIVA reference values for Brazilian HIV+ adolescents, we used healthy Spanish adolescents as the reference population. The sample size also limited our ability to conduct sex-stratified analyses; however, sex was included as a variable in one of the multivariable models to address this limitation, although it did not show a significant association. Finally, the cross-sectional design of this study does not allow for conclusions about causality. 

 In conclusion, this study found a direct association between peak VO_2_ and PhA in HIV+ adolescents. The combination of peak VO_2_ and other covariates explains approximately half of the variations in PhA. Additionally, BIVA revealed alterations in hydration status and a lower classification for BCM and FFM in HIV+ adolescents compared to the reference population. These findings suggest that both PhA and BIVA are valuable tools for health professionals to monitor and track morphological changes in HIV+ adolescents, particularly regarding cellular integrity and overall health. 

## Data Availability

The database that originated the article is available with the corresponding author
